# Development and Characterization of Films Containing Sichuan Pepper Extract to Extend the Shelf Life of Refrigerated Beef Patties

**DOI:** 10.3390/foods14193335

**Published:** 2025-09-25

**Authors:** Inés Mus-León, María Muñoz-Núñez, Juliana Villasante, Idoia Codina-Torrella, María Pilar Almajano

**Affiliations:** 1 Barcelona School of Industrial Engineering, Universitat Politècnica de Catalunya, Av. Diagonal 647, 08028 Barcelona, Spain; ines.mus.leon@upc.edu; 2 Chemical Engineering Department, Universitat Politècnica de Catalunya, Av. Diagonal 647, 08028 Barcelona, Spain; juliana.villasante@upc.edu; 3 Centre d’Innovació, Recerca i Transferència en Tecnologia dels Aliments (CIRTTA), Department of Animal and Food Science, Facultat de Veterinària (Edifici V), Universitat Autònoma de Barcelona, Travessera dels Turons, 08193 Bellaterra, Spain

**Keywords:** Sichuan pepper, gelatin, alginate, film, meat preservation

## Abstract

This study explores innovative approaches for sustainable food preservation by incorporating Sichuan pepper extract into biodegradable gelatin and alginate films. In response to growing environmental and health concerns, these natural polymers offer alternatives to petroleum-based plastics and synthetic additives. The aim of this study was to compare films made from gelatin and alginate and containing Sichuan pepper extract (2.5 and 5%) and to evaluate their effectiveness in extending the shelf life of refrigerated beef patties. Scavenging activity and polyphenol content of the extract were evaluated by DPPH (4.70 µmol TE/mL), ABTS (4.03 µmol TE/mL), and Folin–Ciocalteu assays (2.35 mg GAE/mL). In addition, the physical characteristics of the films were also assessed. Film characterization showed that gelatin-based films had greater stiffness (water-based alginate film; 1156 MPa), which diminished with increased extract content (5% extract-based alginate film: 215.5 MPa), and surface homogeneity also declined with higher extract content. However, higher concentrations of the extract (5%) improved optical properties such as UV protection and opacity. Preservation tests performed on beef patties revealed that the films with the extract could significantly reduce lipid oxidation, with lower TBARS values observed in samples covered with these films. Nevertheless, no significant differences were detected between films with the extract. Moreover, samples without the extract were the most oxidized, proving that the film without the extract had no protective effect against oxidation. Overall, these findings underscore the potential of Sichuan pepper as a natural ingredient and highlight the promise of biodegradable films as an effective and eco-friendly strategy for meat product packaging.

## 1. Introduction

In recent years, the food industry has transformed due to increasing concerns about the environmental and health implications of food production and preservation [1]. This shift has created the need for new sustainable strategies, such as biodegradable films for food preservation fabricated from natural polymers containing plant-based extracts [2,3]. This strategy is not only environmentally friendly, compared to conventional petroleum-based plastics, but it might also help extend the shelf life of food products and slow down processes such as lipid oxidation.

Meat products, like patties, have considerable lipid content, which makes them especially susceptible to oxidative deterioration [4]. Lipid oxidation is a free radical chain reaction, which starts with the rupture of the carbon–hydrogen unsaturated bond, resulting in free radicals [5]. The lipid oxidation process in meat can negatively impact its color, texture, and flavor [6]. Synthetic antioxidants, like butylated hydroxyanisole (BHA) and butylated hydroxytoluene (BHT), have been commonly used to mitigate these effects. However, their potential toxicity has led to consumer rejection [7].

These growing concerns have encouraged scientists to focus on natural alternatives. In this aspect, Sichuan pepper, also known by its scientific name as *Zanthoxylum bungeanum* Maxim, has gained significant interest [8]. This spice is traditionally used in China as an ingredient in gastronomy due to its tingling effects [9]. Furthermore, Sichuan pepper is also known for its antioxidant and antimicrobial activities and for its health benefits, due to its rich flavonoid content [10,11]. Therefore, incorporating Sichuan pepper extract into polymer films represents a promising strategy for food preservation, potentially improving the shelf life of meat products.

Alginate is a polysaccharide derived from brown algae [12]. It has been widely used in different applications due to its capacity to form films and gels [13]. Alginate films can be used in preserving food products [14]. In meat products such as hamburgers, alginate films have been shown to regulate water activity and gas exchange, which are critical factors in delaying spoilage [15]. Additionally, these films have the ability to incorporate oils and extracts from plants with antioxidant or antimicrobial properties, making them valuable as a new meat preservation strategy [16,17]. Moreover, other biodegradable polymers, such as gelatin, have been explored as alternatives. Gelatin films have gained attention for their excellent oxygen barrier properties, making them suitable for packaging high-fat items like meat [18]. Gelatin is obtained from collagen sources [19]. These films are biodegradable and compatible with natural additives and ingredients, and they are highly suitable for incorporating plant extracts, which can improve their properties [20,21].

Different studies have investigated the possibility of including various natural ingredients such as natural extracts or essential oils from plants, which can add beneficial properties to the films. When it comes to gelatin-based films, some studies have showed that incorporating plant-based extract into edible films can elongate the shelf life of fresh meat [22]. For example, rice bran, beetroot peel, and pomegranate peel extracts, among others, have been used, and the results obtained in terms of color and pH maintenance, as well as shelf life extension, are promising [23,24]. As for alginate films, existing research works indicated that phenolic extracts, like essential oils and herb extracts, incorporated into active packaging applications show some antioxidant and antimicrobial activities [25,26]. Although numerous studies have evaluated the combination of different plant-based extracts into alginate and gelatin films, to the best of our knowledge, there is no information on the use of Sichuan pepper extract in alginate and gelatin films in order to improve the shelf life and preservation of beef patties.

Against this backdrop, this study investigates the effect of the incorporation of an extract made from Sichuan pepper into sustainable and edible films. These films are made using biodegradable polymers such as gelatin and alginate with different concentrations of Sichuan pepper extract. The objective of this study is to compare the effectiveness of both films (by integrating the extract at two different concentrations) in extending the shelf life and preserving the quality of beef patties in refrigerated conditions (4 ± 1 °C). Furthermore, it is important to note that the films have been characterized to compare their physical and optical properties. In a commercial environment, bioactive films are expected to be placed below and above beef patties to enhance the effectiveness of the active compounds.

## 2. Materials and Methods

### 2.1. Materials

*Zanthoxylum bungeanum* Maxim, commonly referred to as Sichuan pepper, was utilized in this study and was sourced from China and obtained as dried peppercorns from a local Asian supermarket in Barcelona, Spain. Gelatin sheets from the brand Dr. Oetker were purchased from a local supermarket in the same city. Fresh beef meat (neck cut) was acquired from a local butcher shop in Barcelona, without the addition of antioxidants or other chemical additives. Alginate (Cimalgin 500) was obtained from Vinpai, Saint-Dolay, France. The other chemical reagents used in this study were of analytical-grade and from Sigma-Aldrich, St. Louis, MO, USA.

### 2.2. Extract Preparation

Sichuan peppercorns were ground with an automatic coffee grinder (Bosch, Gerlingen, Germany) and sifted using a steel mesh sieve (CISA, Barcelona, Spain) to obtain a fine powder (particles < 1 mm^3^). The extraction was performed by maceration, using water as a solvent, since previous trials using alcoholic extractions (between 30 and 70%) exhibited poor compatibility with the alginate matrix during film formation. Two different extracts were obtained, i.e., one using a ratio of powder/solvent of 5:100 (*w*/*v*) and another one of 2.5:100 (*w*/*v*). The maceration was performed by stirring for 30 min at 60 °C, to extract higher polyphenolic content [27]. These extracts were used directly to obtain different films.

To express the different results per gram of dry weight, the resulting mixture was cooled and later centrifuged at 3000 rpm for 10 min (Orto Alresa Mod. Consul, Ortoalresa, Ajalvir, Madrid, Spain) to separate the solid particles from the extract. Then, the extract was frozen and weighed.

### 2.3. Preparation of Films

Six different films were prepared as shown in Table 1. Two control films contained water and two films contained two different concentrations of Sichuan pepper aqueous extract (2.5% and 5%). Gelatin films were formulated by dissolving 4% (*w*/*w*) gelatin and 0.8% (*w*/*w*) sorbitol. Alginate films were produced using 1.2% (*w*/*w*) sodium alginate, the corresponding base, and 0.3 g of glycerol per gram of alginate.

All mixtures were poured into Petri dishes (13 cm in diameter), 27 g per dish, and left to dry at room temperature, approximately 23 °C, for three days. In the case of the alginate film, the Petri dishes were first coated with chloromethyl trimethylsilane and subsequently dried, to facilitate film extraction. Once dried and removed, the films were trimmed to match the diameter of the patties (5 cm). Enough films were prepared to ensure that both the top and bottom surfaces of each patty remained in direct contact with the active material.

### 2.4. Antioxidant Activity and Polyphenolic Content of the Extracts

#### 2.4.1. DPPH

DPPH values were determined using the method described by Maqsood et al. [27] with some adaptations. The radical solution was made of 40 mg of DPPH per liter of methanol. It was mixed with the Sichuan pepper extract and, after being kept for 15 min in the dark, the absorbance was determined at 519 nm with the micro-plate reader FLUOstar^®^ Omega (V2, BMG LABTECH, Ortenberg, Germany). The antioxidant capacity was calculated by using Equation (1) [28]:

(1) $$\%DPPH\;decreasing=\frac{Initial\;absorbance-Final\;absorbance}{Initial\;absorbance}\times 100$$

DPPH values were determined from a calibration curve made with Trolox at different concentrations (15 to 500 μM) (R^2^ = 0.998). Results are expressed in millimoles of Trolox equivalents per gram of dry weight (mM TE/g DW).

#### 2.4.2. ABTS

This procedure was adapted from the protocol established by Re et al. [29], with minor modifications. Initially, a 5 mM ABTS radical solution was combined with a 2.5 mM potassium persulfate solution in a 2:1 ratio. The resulting ABTS stock solution was stored in the dark at 25 °C for 16 h. Before use, the stock solution was diluted with milliQ water to a solution with an absorbance between 0.9 and 1.1 that was determined at 734 nm with the micro-plate reader FLUOstar^®^ Omega (V2, BMG LABTECH, Ortenberg, Germany). Then, the extract was added, and the absorbance was measured after 15 min. The results are expressed as millimoles of Trolox equivalents per gram of dry weight (mM TE/g of DW).

#### 2.4.3. Determination of Total Polyphenolic Content

The determination of total polyphenols was carried out by the Folin–Ciocalteu reagent method described by Villasante et al. [30]. The results are expressed as milligrams of gallic acid equivalent per mL (mg GAE/mL), based on a calibration curve made with gallic acid (100–1700 µM, R^2^ = 0.992).

### 2.5. Film Characterization

#### 2.5.1. Transmittance

Firstly, the films were cut into pieces (1 × 2.5 cm) and placed on the inner side of a quartz cuvette. Then, the transmittance of six different films was obtained after scanning with the UV spectrophotometer SPECTROstar Nano (BMG LABTECH, Ortenberg, Germany) in the range of 220–1000 nm.

The opacity of the film was calculated by the following Equation (2) [31]:

(2) $$value=\frac{{-logT}_{600}}{x}$$

where T_600_ is the fractional transmittance at 600 nm, and x is the equivalent film thickness (mm), measured using a micrometer (Mitutoyo, Elgoibar, Spain). The lower opacity value indicates the higher transparency of films. The results were carried out as duplicates.

#### 2.5.2. Morphology and Microstructure of Films

Scanning Electron Microscopy (Nano Nova 230, FEI, Hillsboro, OR, USA) was used to characterize the surface microstructure of the films. The films were cut (2 mm), mounted onto stubs, and sputter-coated with graphite. The surface microstructure of the films was observed at a voltage of 5 kV and a magnification of ×100.

#### 2.5.3. Molecular Structures and Functional Groups

Molecular structures and functional groups were characterized using a Fourier-transform infrared spectrometer (Spectrum Two, PerkinElmer, Waltham, MA, USA). The films were scanned at wavelengths ranging from 500 cm^−1^ to 4000 cm^−1^. Each sample was scanned 64 times at a resolution of 4 cm^−1^.

#### 2.5.4. Mechanical Properties

The mechanical properties of the films were evaluated in terms of elongation at break, tensile strength, and Young’s modulus. A tensile testing machine (ZwickRoell, Ulm, Germany) equipped with a 5 kg load cell (probe S/N 778312) was used. Before tests, films were cut into probes with a width of 2.24 mm, and the thickness was also measured with a micrometer (Mitutoyo, Spain). Tensile strength and elongation at break were obtained as the maximum stress reached before rupture and as the strain at the breaking point. Young’s modulus was determined as the slope of the initial linear region of the stress–strain curve.

### 2.6. Meat Preservation

#### 2.6.1. Beef Patties

**Figure 1:**
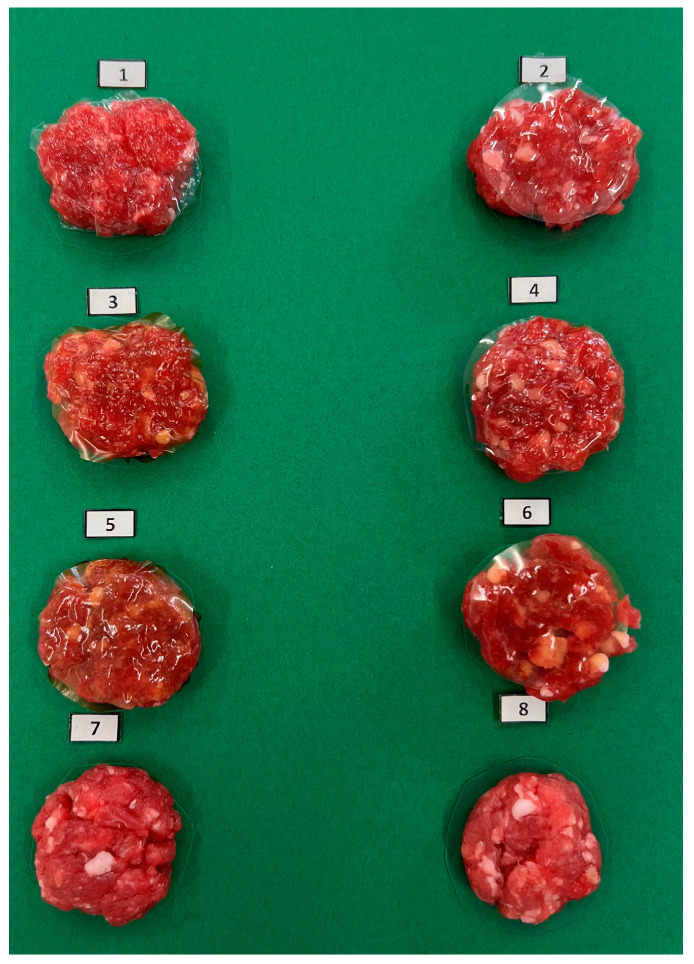
Beef patties with eight different treatments: (1) AA meat covered with the alginate film without extract; (2) GA covered with the gelatin film without extract; (3) A2.5 covered with the alginate film with 2.5% extract; (4) G2.5 covered with the gelatin film with 2.5% extract; (5) A5 covered in 5% extract alginate film; (6) G5 covered in 5% extract gelatin film; (7) C−, negative control; and (8) C+, positive control. Patty samples were captured using a tripod-mounted iPhone 13 Pro camera (Apple, Cupertino, CA, USA), placed at a fixed distance of 30 cm from the samples. A white LED lamp (6000 K, 2000 lumen) was used as a light source, located at 30 cm from the samples, to provide uniform illumination. A neutral green background was employed to avoid reflections and ensure consistency across all images.

Patties were made with minced beef as shown in Figure 1. The meat was mixed well manually with salt (1%, *w*/*w*) and divided into eight parts for each treatment: AA (covered with the alginate film without extract), A2.5 (covered with the alginate film with 2.5% extract), A5 (covered in 5% extract alginate film), GA (meat covered with the gelatin film without extract), G2.5 (covered with gelatin film with 2.5% extract), G5 (covered in 5% extract gelatin film), C− (negative control, meat without additives), and C+ (positive control, meat with 1% (*w*/*w*) of commercial mix of additives (corn starch, dextrose, sodium sulfite, sodium ascorbate, and trisodium citrate, ref.4409, Mane, Barcelona, Spain). For each treatment, patties of 8 g with a 5 cm diameter were made, placed in trays at 4 ± 1 °C, and preserved in refrigerated conditions (4 ± 1 °C) for 7 days. On days 0, 3, 4, 5, 6, and 7, various parameters were analyzed.

Patties were covered with alginate and gelatin edible films placed on both top and bottom surfaces to ensure direct contact with the meat and evaluate the protective role of these films. Moreover, patties were placed in plastic trays covered with a layer of cling film and stored at 4 ± 1 °C for 7 days.

#### 2.6.2. pH

pH was measured directly in the patty samples using a digital pH meter with a penetrating probe (YK-25, Yinmic, Shandong, China). The pH meter was carefully calibrated before each use with a buffer solution of pH 4 and pH 7.

#### 2.6.3. Metmyoglobin

Following the method described by Gallego et al. [32], 1 g of the patty sample was homogenized in 5 mL phosphate buffer, 0.04 M with pH 6.8, for 30 s, using an ultraturrax (Ika-Werke, GmbH & Co., Staufen, Germany). Then, it was centrifuged at 4000 rpm for 10 min. Lastly, the supernatant absorbance was measured at 525, 545, 565, and 572 nm. The percentage of metmyoglobin was determined using Equation (3):

MetMb% = [2.514(A_572_/A_525_) + 0.777(A_565_/A_525_) + 0.8(A_545_/A_525_) + 1.098] × 100 (3)

#### 2.6.4. Color (CIELab)

Color was determined for all the samples using a Minolta colorimeter CR-400 (Konica Minolta, Tokyo, Japan) (equipped with a pulsed xenon lamp, illuminant D65, with an observer angle of 10°). It was expressed using CIELab parameters with L* (luminosity), a* (positive, redness/negative, greenness), and b* (positive, yellowness/negative, blueness) values.

#### 2.6.5. Microbiology

Aerobic mesophilic total counts of the patties on days 0, 4, and 7 were determined following Villasante et al. [30]. Multiple test tubes were filled with 4.5 mL of Ringer’s solution, which was prepared by dissolving one tablet per 500 mL of water. Approximately 5 g (m) of the patty samples was homogenized with 9 mL of Ringer’s solution using a stomacher. From this homogenized solution, 0.5 mL was transferred (dilution: −1) into the first test tube and mixed with a vortexer. Subsequent dilutions were prepared by transferring 0.5 mL from one tube to the next. Then, 0.1 mL of each was inoculated onto Petri dishes. To determine the mesophilic bacteria, the dishes were incubated at 30 °C for 48 h (ISO 4833-1, 2013) [33]. The results are expressed as the base 10 logarithm of the number of colony-forming units per gram of sample (log CFU/g).

#### 2.6.6. Fatty Acid Content

The fatty acid profile of all the samples was determined using the extraction method according to Folch et al. [34]. A GC-2025 with an autosampler (Shimadzu, Kyoto, Japan) fitted with a flame ionization detector and a capillary column (L × I.D. 30 m × 0.25 mm, df 0.25 µm) (Trajan Scientific and Medical, Ringwood, Australia) was used for the analysis in accordance with Villasante et al. [35]. After being kept at 60 °C for one minute, the oven temperature was increased to 260 °C at a rate of 6 °C per minute. The temperatures of the detector and injector (AOC-20i, Shimadzu, Kyoto, Japan) were set at 280 °C and 260 °C, respectively. Helium was used as a carrier gas, and a split ratio of 1:20 was injected into the sample. Fatty acids were identified by comparing the FAME retention times with the Supelco 37 Component FAME Mix (Sigma-Aldrich, St. Louis, MO, USA). Three replicate GC analyses were performed, and the results were expressed in GC area % as mean values ± SD.

#### 2.6.7. Lipid Oxidation

The lipid oxidation was evaluated by determining thiobarbituric acid reactive substances (TBARSs). In a test tube, 1 g of the meat sample was mixed with 0.5 mL of EDTA (0.3%) to stop oxidation. Then, 2.5 mL of TCA (10% HCl, 0.25 M) were added and homogenized with an ultraturrax (Ika-Werke, GmbH & Co., Staufen, Germany). The mixture was centrifuged at 4000 rpm for 10 min. The liquid solution was mixed with thiobarbituric acid (TBA) in a 1:1 ratio (*v*/*v*). Subsequently, the mixture was heated at 90 °C for 10 min and then cooled to room temperature. The absorbance was measured at 532 nm. The results were expressed as milligrams of malondialdehyde (MDA) per kilogram of meat sample (mg MDA/kg meat sample).

### 2.7. Statistical Analysis

The results were statistically analyzed using the Minitab^®^ 17 software (Minitab, Inc., State College, PA, USA) by analysis of variance (ANOVA), and Tukey’s multiple comparison test was applied to determine the significant differences among samples (*p* < 0.05). The measurements were carried out in triplicate (those expressed as mean value ± standard deviation).

## 3. Results and Discussion

### 3.1. Radical-Scavenging Activity and Polyphenolic Content of Sichuan Pepper Extract

Results obtained by DPPH and ABTS methods corresponded to 4.70 μmolTE/mL of the extract and 4.03 μmolTE/mL of the extract, respectively.

These values indicate high radical-scavenging activity and provide a robust preliminary assessment that confirms the radical-scavenging potential of the samples, which could also be effective in non-aqueous media.

The results obtained using the Folin–Ciocalteu method (around 2.35 mg GAE/mL) confirm the presence of phenolic compounds in the sample, which supports the radical-scavenging potential of the extracts and their subsequent use in the preparation of the bioactive films. Previous research has reported a wide diversity of phenolic compounds in Zanthoxylum bungeanum. The identification of polyphenols in this species has expanded from about 40 to over 150 compounds [36]. Among these, the major polyphenolic components include chlorogenic acid, hyperoside, quercitrin, quercetin-3-arabinoside, isorhamnetin-3-glucoside, and syringetin-3-glucoside. The presence of such compounds, many of which are well-known antioxidants, is in line with the high radical-scavenging activity observed in this study [37].

### 3.2. Results Obtained from Film Characterization

To characterize the films, various analyses were conducted, such as assessing optical absorbance in the visible and UV range, surface morphology via electron microscopy, key spectral bands with Fourier-transform infrared spectroscopy (FTIR), and mechanical strength through Young’s modulus. These techniques were used to compare the different films and determine how the addition of Sichuan pepper extract may affect film characteristics.

#### 3.2.1. Visual Aspect of Alginate and Gelatin Films

**Figure 2:**
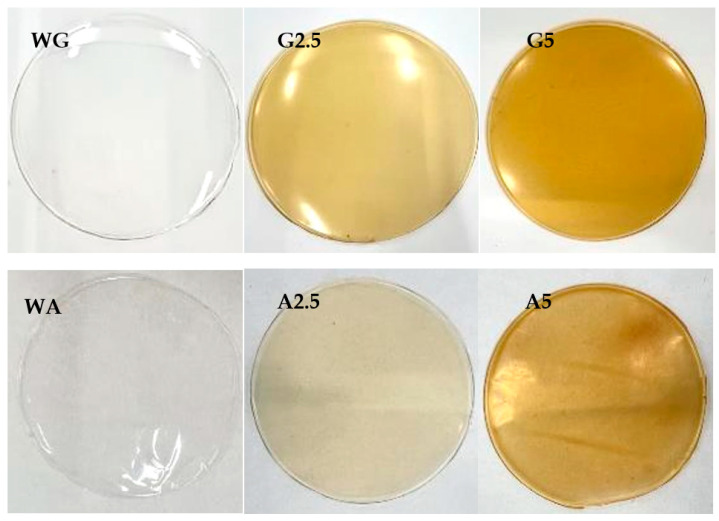
Alginate and gelatin films: (WG) water-based, (G2.5) 2.5% extract-based, and (G5) 5% extract-based, (WA) water-based, (A2.5) 2.5% extract-based, and (A5) 5% extract-based.

Figure 2 shows the images of the final films. The control film without the extract appears transparent, whereas films containing the extract exhibit a progressively more intense yellow-brown coloration as the extract concentration increases. In addition, the extract seems to be better dispersed in the gelatin-based films than in the alginate-based ones. For instance, the film G5 (gelatin with the highest extract concentration) appears more homogeneous compared with the alginate film when adding the same amount of extract (A5).

#### 3.2.2. Light Transmission and Opacity of Films

**Figure 3:**
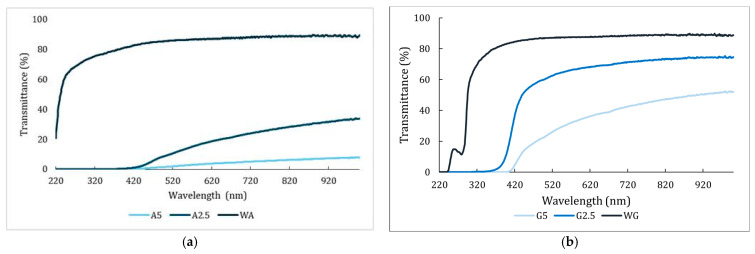
UV and visible range transmittance of (**a**) alginate and (**b**) gelatin films. A5: alginate film with 5% Sichuan pepper extract; A2.5: alginate film with 2.5% Sichuan pepper extract; WA: water-based alginate film; G5: gelatin film with 5% Sichuan pepper extract; G2.5: gelatin film with 2.5% Sichuan pepper extract; GA: water-based gelatin film.

Studying the light transmission and opacity of films in contact with food is important since they can protect food products from the effects of light and UV radiation [38]. Figure 3 shows the three different films of (a) alginate and (b) gelatin spectral transmittance within UV and visible light regions.

The addition of Sichuan pepper extract reduced the transmittance in all films. Films containing the extract (A5, A2.5, G5, and G2.5) showed no transmittance below 400 nm. This indicated a protective effect by blocking UV radiation [39]. This effect was singly weaker in the case of G2.5, since it showed a transmittance of 13.7% at 400 nm. Moreover, the alginate films exhibited lower visible light transmittance (400–800 nm) than the gelatin films at the same extract levels. This difference may be attributed to the interaction between the extract compounds and the film matrix. Similar findings have been reported by Li et al. [40], who suggested that differences in light barrier properties can result from variations in film thickness, extract concentration, and the interaction of bioactive compounds with the polymer. In the case of alginate films, A2.5 reached a 27.47% light transmittance at 800 nm, while the film with 5% extract (A5) displayed the lowest transmittance values (5.97%). Contrarily, for gelatin films, the light transmittance was higher; G2.5 showed a transmittance of 46.22% and G5 reached 72.95% at 800 nm. The reduced transmittance was observed when the extract concentration increased, which could be attributed to phenolic compounds and natural pigments being present in the extract that can absorb visible light. Similar results have been observed in other studies on alginate and gelatin films with natural extracts such as papaya peel and murta ecotype leaves [31,41].

The results of the opacity of the film were in line with those observed for the light transmittance behavior. Increasing the concentration of Sichuan pepper extract resulted in higher opacity values for both gelatin and alginate films. These results were similar to those obtained by Huo et al. [42] with the increase in the amount of green Sichuan pepper essential oil. However, alginate films exhibited greater opacity than gelatin films (*p* < 0.05). A5 and A2.5 showed the highest opacity values, with values of 14.16 ± 3.27% and 9.31 ± 1.19%, respectively. These values were significantly higher (*p* < 0.05) than those observed in gelatin films with the same extract concentrations, which reached opacities of 2.94 ± 0.98% and 1.24 ± 0.36%, respectively. In contrast, films without the extract exhibited the lowest opacity values. No statistically significant differences (*p* > 0.05) were observed between the gelatin control films and the gelatin films containing the extract (G5 and G2.5). WA and WG had an opacity of 1.23 ± 0.17% and 0.49 ± 0.07%, respectively. Crizel et al. [31] have presented studies using a papaya powder at two different concentrations, 2.5% and 5%. The opacity values obtained were similar to those reported in this study. An opacity of 0.73% was obtained at a concentration of 2.5% and 1.14% when a concentration of 5% was added to the gelatin films [31]. On the other hand, Li et al. [38], working with natural extracts obtained from green tea, ginkgo leaf, and ginger, reported a concentration of 1.0 mg/mL in the extract, which could be equivalent to obtaining an extract at 0.1% lower than those used in this study. This leads them to obtain lower opacity values (0.540–0.570%) [38]. Alginate films have been reported to have greater opacity when adding 0.5 g/mL ginseng extract (1.06–1.39%) or when adding 50% of green tea (3.10%) [40,43]. These differences may be related to the dissolution behavior of the Sichuan pepper extract in the alginate and gelatin matrices and their different affinities for interactions before film formation. These findings suggested the potential of films combined with Sichuan pepper to enhance opacity and UV protection and prevent oxidation processes in meat.

#### 3.2.3. Findings of Morphology and Microstructure of Films

**Figure 4:**
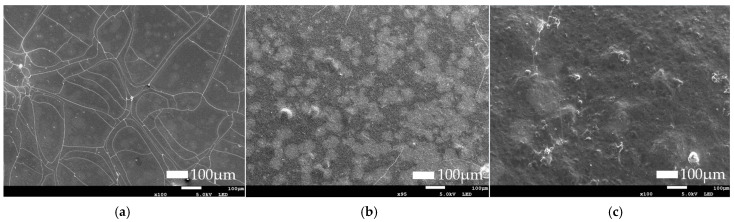
SEM of alginate film: (**a**) water-based alginate film (WA); (**b**) 2.5% (*w*/*v*) Sichuan pepper extract-based alginate film (A2.5); (**c**) 5% (*w*/*v*) Sichuan pepper extract alginate film (A5).

**Figure 5:**
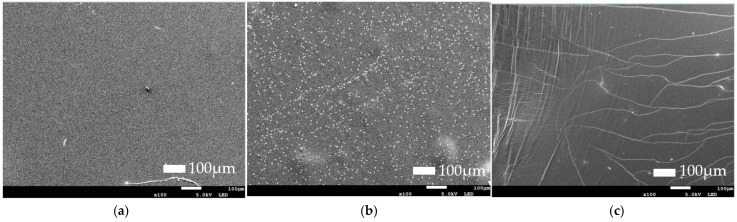
SEM of gelatin film: (**a**) water-based gelatin film (WG); (**b**) 2.5% (*w*/*v*) Sichuan pepper extract-based gelatin film (G2.5); (**c**) 5% (*w*/*v*) Sichuan pepper extract gelatin film (G5).

Figure 4 and Figure 5 show the surface microstructure of alginate and gelatin films, respectively. For alginate films, the film without the extract (WA) showed a more fragile structure than the films with the extract (A2.5 and A5), as small cracks could be observed. These fractures in the internal structure can occur when the film is detached from the Petri dish. At a lower concentration of extract (A2.5), the film exhibited a smooth and homogeneous surface. In contrast, the film with the highest extract concentration (A5) showed a less uniform surface with more imperfections that could potentially correspond to an aggregation of the extract. Similar results were obtained by Villasante et al. where adding higher concentrations of pecan walnut extract led to a more heterogeneous surface in films [44]. The same was observed by Wang et al. [45] when increasing the *Zanthoxylum armatum* DC concentration from 0.5% to 2.5%.

Li et al. [38] suggested that higher concentrations may hinder the dissolution of the extract in the matrix, and this may generate a more heterogeneous surface. This could contribute to lower visible light transmittance, as was observed in Section 3.2.2. However, G5 showed a network of lines, which may indicate internal stress, which could be due to incompatibility between the extract and gelatin matrix [46]. This morphology suggests that, at higher concentrations, the compounds in the extract may interfere with the normal formation of the three-dimensional gelatin network during the gelation process. In the G2.5 film, numerous spherical particles were observed, distributed moderately throughout the gelatin matrix. This may suggest an agglomeration of particles that do not dissolve properly.

#### 3.2.4. Mechanical Resistance of the Films

Young’s modulus is used to evaluate the mechanical resistance of the films. Its values can vary depending on factors such as film thickness, crystalline structure, porosity, and residual stress. Table 2 presents the values for Young’s modulus and film thickness across the samples.

Significant differences (*p* < 0.05) were observed depending on the type of film and the concentration of Sichuan pepper extract added. For Young’s modulus (E), the general trend was that increasing the concentration of extract led to a decrease in stiffness for both alginate- and gelatin-based films. This effect has also been reported by Villasante et al. [44] who proposed that increasing the extract concentration might increase the interactions of the extract compounds with the polymer chains. Hosseini et al. [47] reported that polyphenols can interact with gelatin chains through hydrogen bonding (between the –OH groups of polyphenols and the –COOH or –NH_2_ groups of gelatin); hydrophobic interactions (between the aromatic rings of polyphenols and hydrophobic amino acid residues); and electrostatic interactions, where positively charged protein groups interact with negatively charged hydroxyl groups of polyphenols, forming a type of crosslink. Such interactions can disrupt the regular arrangement of the polymer matrix, resulting in films with lower stiffness. In contrast, the maximum tensile strength (σ max) was generally higher for gelatin-based films than for alginate films, with WG showing the highest value (46.45 MPa). These results are in line with a study that compared characteristics of both alginate and gelatin films when adding different concentrations of polyethylene glycol. The results of this study showed that the films made from gelatin polymer had a greater stiffness compared to alginate films [48]. However, the addition of 5% extract weakened the films, reducing their tensile strength. Additionally, these two parameters might be influenced by film thickness (h). Gelatin films (WG and G2.5) were significantly thicker than alginate films, which could contribute to their higher σ max and lower E. On the other hand, the elongation at the breaking point (ε max) did not show significant differences between the different samples, except for the gelatin gel, which increased with the addition of 2.5% extract. This suggests that a moderate amount of extract may act as a plasticizer, improving deformability. In contrast, adding 5% extract reduced elongation, making the films more fragile and less ductile.

#### 3.2.5. Identification of Molecular Structures and Functional Groups

**Figure 6:**
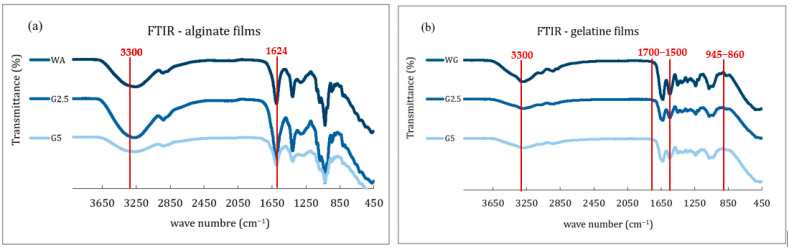
Fourier-transform infrared spectroscopy: (**a**) alginate film spectrum; (**b**) gelatin film spectrum. The red lines highlight the most notable spectral changes.

FTIR is a technique used to identify molecular structures and functional groups in a sample by analyzing how it absorbs infrared radiation. The FTIR spectrum presented in Figure 6 showed the infrared transmittance patterns of the different film samples composed of alginate and gelatin matrices, each containing different concentrations of Sichuan pepper extract. These spectra revealed changes in intermolecular interactions influenced by the polymer type.

For both alginate- and gelatin-based films, the most notable spectral changes occur in the regions corresponding to O–H stretching centered at around 3300 cm^−1^. This peak may suggest a possible hydrophilic interaction or water retention. Additionally, when it comes to alginate-based films, at around 1624 cm^−1^, the C = O and asymmetric COO^−^ stretching bands may point to interactions between the extract and carboxylate groups. On the contrary, the gelatin-based films presented the characteristic amide I and II bands, between 1700 and 1500 cm^−1^, associated, respectively, with C = O and C–N stretching and N–H bending, confirming the protein structure of gelatin [49]. Finally, the presence of glycerol may also be identified by a band at 945 cm^−1^ and at 860 cm^−1^, corresponding, respectively, to O–H and C–O stretching, located within the fingerprint region between 1100 and 800 cm^−1^ [50]. Moreover, the addition of the plant extract at different proportions did not alter the structural composition of the base film.

### 3.3. Meat Quality and Preservation

The evaluation of the alginate- and gelatin-based films with varying concentrations of Sichuan pepper extract was carried out by analyzing the behavior of beef patties, protected by these films, stored under refrigeration at 4 ± 1 °C for 7 days. Periodic monitoring of aerobic mesophilic total bacteria, degree of oxidation, pH, color, and metmyoglobin concentration was performed.

#### 3.3.1. pH Determination

pH is an indicator that plays a key role in meat quality, affecting texture, color, water retention, and microbial growth. The evolution of pH for all the samples over the 7 days is presented in Table 3. On day 0, all samples presented the same value of pH (*p* > 0.05) (Table 3). A progressive increase in pH was observed for all samples during storage time, with final values ranging between 6.10 and 7.00. This rise in pH may be due to proteolysis produced by microbial activity, since during this process, metabolites such as amines and ammonia are formed and they can increase the pH of the meat [51,52]. A rise in pH was observed in both patties with alginate and gelatin films, although samples coated with gelatin-based films generally exhibited a more pronounced pH rise (Table 3). On the other hand, it was also observed that, the higher the extract concentration, the greater the pH increase. For instance, G5 showed the highest pH value (~7.00) on day 7. Other studies have already observed that the addition of natural extracts or essential oils in films increased the pH of the meat [53,54].

Among all treatments, C+ showed the least pH variation, reaching only 6.03 on day 7, likely due to the inhibitory effects of antioxidants on microbial activity and proteolysis.

#### 3.3.2. Metmyoglobin and Color

**Figure 7:**
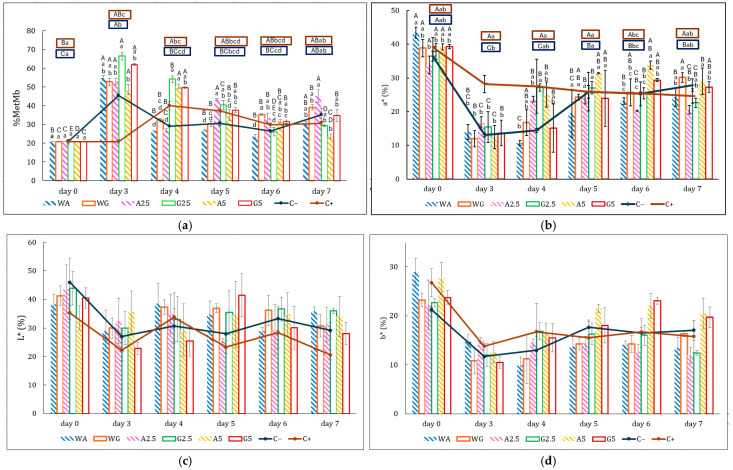
Evolution of metmyoglobin and color in patties with eight different treatments: (C−) negative control, patty without additives; (C+) positive control, patty with commercial preparation; (A5) patty covered in 5% extract-based alginate film; (A25) patty covered in 2.5% extract-based alginate film; (WA) patty covered in water-based alginate film; (G5) patty covered in 5% extract-based gelatin film; (G25) patty covered in 2.5% extract-based gelatin film; (WG) patty covered in water-based gelatin film. The four figures correspond to (**a**) evolution of metmyoglobin content; (**b**) evolution of color parameter L*; (**c**) evolution of color parameter a*; and (**d**) evolution of color parameter b*. ^A–E^ Mean values with different superscripts within the same treatment but at different times are statistically different; ^a–d^ mean values with different superscripts on the same day but from different treatments are statistically different (*p* < 0.05).

**Figure 8:**
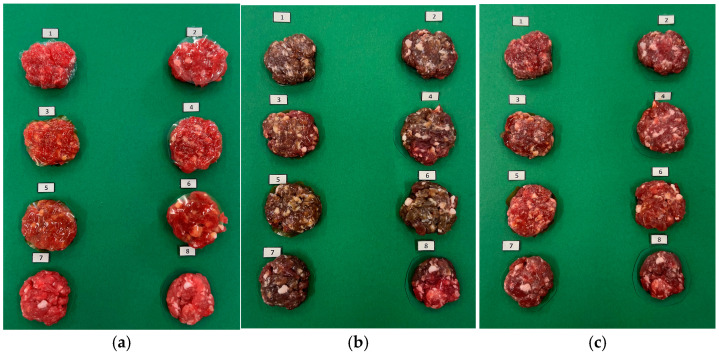
Visual evolution of color in patties on different days: (**a**) initial day; (**b**) day 3; (**c**) last day (day 7). Patty samples were captured using a tripod-mounted iPhone 13 Pro camera, placed at a fixed distance of 30 cm from the samples. A white LED lamp (6000 K, 2000 lumen) was used as a light source, located at 30 cm from the samples, to provide uniform illumination. A neutral green background was employed to avoid reflections and ensure consistency across all images.

Initially, fresh beef has a purplish-red color (Figure 7 and Figure 8) due to the presence of deoxymyoglobin. When exposed to oxygen, it converts to oxymyoglobin, which gives meat a bright red look. Over time, oxymyoglobin can oxidize to form metmyoglobin, resulting in the meat having a brown color. Therefore, metmyoglobin content is an oxidation indicator of meat [55]. The evolution of meat color and metmyoglobin in samples covered with films containing Sichuan pepper extract is shown in Figure 7.

During the first three days, a* (redness) values decreased for all the samples, accompanied by a sharp increase in metmyoglobin content (Figure 7b). Moreover, b* and L* values decreased, thus giving meat a brown color. This early deterioration in color can be attributed to the exposure to air before storage. Oxymyoglobin is highly unstable and rapidly oxidizes to metmyoglobin, resulting in visible discoloration and declining values of a*, b*, and L* [55]. However, the positive control (C+) exhibited a delayed oxidation pattern, with significantly lower metmyoglobin levels until day 4 (Figure 7a).

On day 4, WG, WA, and A2.5 showed a slight decrease in metmyoglobin. Some authors explain that the increase in metmyoglobin may be due to microbial activity capable of reducing metmyoglobin to deoxymyoglobin [56]. These results would justify an increase in b* and L* values to give more purple colors. Following this point, metmyoglobin levels tended to stabilize for all samples. On day 7, samples G5 and A5 were the samples with the lowest metmyoglobin content.

Lastly, the stabilization of the color may be eventually stopped by enzymatic degradation. As reductase enzymes lose activity over time, a secondary rise in metmyoglobin is observed, leading to mild color deterioration once again. Similar post-stabilization trends are reported by Zhang S (2023) [57]. A more visual representation of these color results is provided in Figure 8.

#### 3.3.3. Evolution of Bacterial Growth

**Figure 9:**
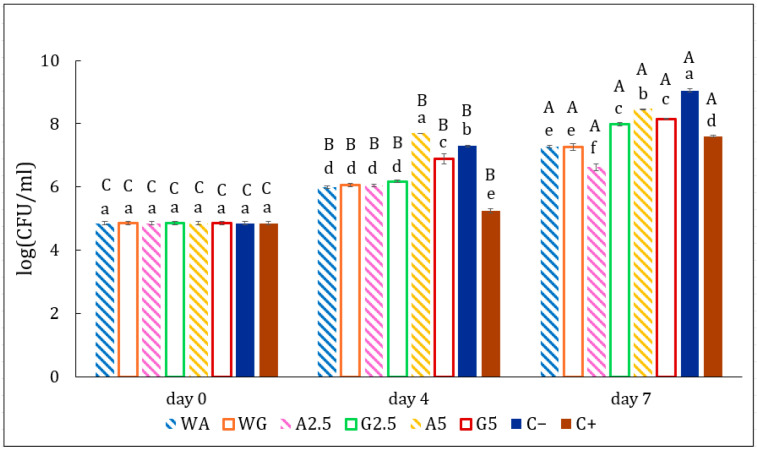
Aerobic mesophilic total counts in patties with eight different treatments: (C−) negative control, patty without additives; (C+) positive control, patty with commercial preparation; (A5) patty covered in 5% extract-based alginate film; (A25) patty covered in 2.5% extract-based alginate film; (WA) patty covered in water-based alginate film; (G5) patty covered in 5% extract-based gelatin film; (G25) patty covered in 2.5% extract-based gelatin film; (WG) patty covered in water-based gelatin film. ^A–C^ Mean values with different superscripts within the same treatment but at different times are statistically different; ^a–f^ mean values with different superscripts on the same day but from different treatments are statistically different (*p* < 0.05).

Figure 9 assesses the aerobic mesophilic total count evolution of meat samples during storage. All samples began with an initial bacterial load of about 5 log CFU/g. By day 7, the negative control reached 10^9^ CFU/mL, whereas samples coated with films remained below 8 log CFU/g. Particularly effective was the alginate film containing 2.5 g of Sichuan pepper extract per 100 mL of water, which recorded the lowest microbial count at 6.7 log CFU/g. Films demonstrated an inhibitory effect on microbial growth. This is because they prevent the transfer of oxygen and carbon dioxide. This has already been observed in different studies testing the efficacy of biodegradable films with or without extracts for the prevention of bacterial growth [53,58].

#### 3.3.4. Lipid Profile of the Patties

When it comes to lipid oxidation, polyunsaturated fatty acids (PUFAs) are the most vulnerable to oxidation because of their multiple double bonds. Therefore, monitoring PUFA levels is an effective method for assessing lipid oxidation during storage.

The data presented in Table 4 demonstrate the decline in lipid profile of the samples on the first and last experimental days. The PUFA levels from the negative control sample dropped from 2.53% on the initial day to 2.04% by the last day. In contrast, for the samples coated with films containing different concentrations of Sichuan pepper extract, most maintained relatively stable PUFA levels, suggesting the potential antioxidant activity of the extract. The exception was the alginate-based film containing 2.5% extract, where PUFA content decreased to 1.77%, indicating less effectiveness in that case.

#### 3.3.5. Secondary Lipid Oxidation

**Figure 10:**
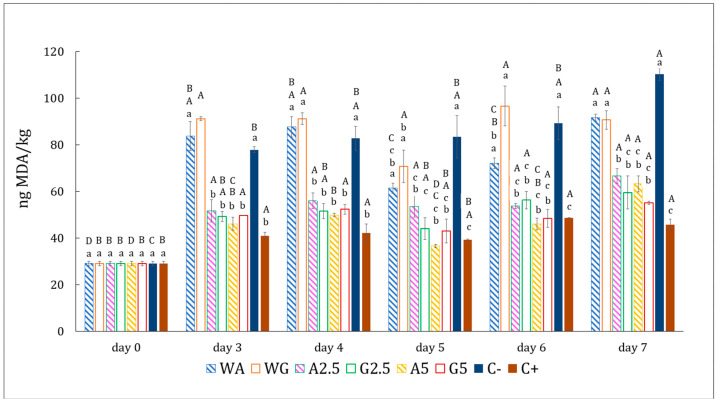
Evolution of TBARSs in patties with eight different treatments: (C−) negative control, patty without additives; (C+) positive control, patty with commercial preparation; (A5) patty covered in 5% extract-based alginate film; (A25) patty covered in 2.5% extract-based alginate film; (WA) patty covered in water-based alginate film; (G5) patty covered in 5% extract-based gelatin film; (G25) patty covered in 2.5% extract-based gelatin film; (WG) patty covered in water-based gelatin film. ^A–D^ Mean values with different superscripts within the same treatment but at different times are statistically different (*p* < 0.05); ^a–c^ mean values with different superscripts on the same day but from different treatments are statistically different (*p* < 0.05) with *n* = 3.

The TBARS assay is a widely used technique to evaluate the formation of aldehydes (as malondialdehyde) present in meat [59]. Figure 10 illustrates the variation in TBARS values of hamburger samples in contact with the films with different concentrations of Sichuan pepper extract, stored at 4 ± 1 °C over a period of 7 days.

For all samples, TBARSs increased over time. The most notable rise occurred on day 3, which was likely the point at which more primary oxidation products (hydroperoxides) were being converted into secondary oxidation compounds (aldehydes, ketones, alcohols, and other volatile compounds) [60]. For the rest of the days, the values remained relatively stable, except for the negative control, which showed a slight additional increase between days 4 and 7 (*p* < 0.05). Samples C−, WG, and WA were the most oxidized, and it is important to note that there were no significant differences between them. This implies that the film alone has no protective effect against oxidation and that the presence of the extract is necessary. Its oxidation is the fastest (among all treatments). All films with the extract have a protective effect against oxidation. However, no significant differences were detected between them, either in terms of concentration or material (gelatin, alginate). This is because there is no linear correlation between concentration and protection against oxidation, but rather it is necessary to reach a point at which it protects and, from there, it provides the same protection unless the concentration is greatly increased (a topic which was not the subject of this study). However, a trend can be observed, i.e., less oxidation in films with higher extract concentrations (5%, A5 and G5). This may be because the concentration used is not different enough to produce a greater effect, as mentioned above. Pinto et al. reported similar results when cinnamon essential oil was added to alginate films in two concentrations (0.1 and 0.05%), and no significant differences in TBARSs were observed between beef burgers. It should be noted that the concentrations reported refer to the total concentration of essential oil in the film, while in this study, we talk about the concentration used to prepare the infusion; hence, they are essentially similar concentrations [61]. On the other hand, in a previous study comparing the effectiveness of alginate and gelatin films in reducing oxidation measured by TBARS values in pastirma (cured meat), it was observed that gelatin films were slightly more effective in preventing lipid oxidation [62]. In contrast, in this study, no significant differences were observed between the two film bases. This result is consistent with the fact that no differences were found in the opacity of the films and, therefore, their barrier properties against UV radiation were similar. All this suggests that the Sichuan pepper incorporated in films may inhibit radical formation and slow down lipid oxidation in meat. The similar antioxidant effectiveness of both concentrations implies that 2.5% may be sufficient.

These findings support the idea that the water-based film alone may not be adequate for oxidative protection and incorporating Sichuan pepper extract into the films might be helpful in reducing MDA formation.

## 4. Conclusions

Sichuan pepper extract showed significant radical-scavenging activity, supported by DPPH and ABTS radical assays and moderate polyphenol levels, proving its potential as a natural source of bioactive compounds for food applications. When incorporated into gelatin and alginate films, its characterization confirmed structural interactions with polymer matrices, improved optical properties like UV protection and opacity, reduced stiffness, and some surface homogeneity loss with higher extract concentrations. In addition, when it comes to film characterization, gelatin-based films were significantly stiffer than alginate-based films, making gelatin films very adequate for food packaging. The successful development of gelatin and alginate films incorporating the extract demonstrates promising potential for use in natural meat preservation. Preservation tests in meat revealed that films with the extract effectively reduced lipid oxidation in beef patties under refrigeration, with no significant changes in pH, color, or metmyoglobin. Interestingly, despite their lower polyphenol content, films made with a less concentrated extract had better results for meat preservation and physical stability in both types of films. These results support the possibility to use Sichuan pepper-enriched biodegradable films as a sustainable packaging strategy for extending shelf life and maintaining quality in beef patties.

## Figures and Tables

**Table 1:** Different films.

WA	Water-based alginate film
WG	Water-based gelatin film
A2.5	2.5% extract-based alginate film
G2.5	2.5% extract-based gelatin film
A5	5% extract-based alginate film
G5	5% extract-based gelatin film

**Table 2:** Young’s modulus trough in tensile test at constant thickness b = 2.24 mm.

Film *	E (MPa)	σ Max (MPa)	ε Max (%)	h (mm)
WA	375.0 ± 49.50 ^b^	8.95 ± 1.63 ^b^	7.55 ± 0.07 ^b^	0.075 ± 0.004 ^b^
A2.5	495.0 ± 50.91 ^b^	15.65 ± 1.34 ^b^	6.70 ± 0.56 ^b^	0.082 ± 0.009 ^b^
A5	231.0 ± 27.90 ^b^	10.05 ± 4.17 ^b^	9.05 ± 5.59 ^b^	0.089 ± 0.001 ^b^
WG	1156.0 ± 302.64 ^a^	46.45 ± 3.75 ^a^	10.05 ± 1.77 ^b^	0.119 ± 0.009 ^a^
G2.5	581.0 ± 59.39 ^b^	38.0 ± 0.99 ^a^	30.80 ± 0.71 ^a^	0.125 ± 0.005 ^a^
G5	215.5 ± 9.19 ^b^	5.90 ± 0.21 ^b^	4.50 ± 0.14 ^b^	0.067 ± 0.003 ^b^

* (A5) 5% extract-based alginate film; (A2.5) 2.5% extract-based alginate film; (WA) water-based alginate film; (G5) 5% extract-based gelatin film; (G2.5) 2.5% extract-based gelatin film; (WG) water-based gelatin film; (E) Young’s modulus; (σ) tensile strength; (ε) elongation at break. ^a,b^ Mean values with different superscripts are statistically different (*p* < 0.05).

**Table 3:** Evolution of pH in patties with eight different treatments (mean value ± standard deviation).

Sample *	Day 0	Day 3	Day 4	Day 5	Day 6	Day 7
WA	5.44 ± 0.05 ^aC^	5.52 ± 0.02 ^bcC^	5.60 ± 0.01 ^cC^	5.90 ± 0.06 ^bcAB^	5.81 ± 0.13 ^cdB^	6.10 ± 0.10 ^eA^
WG	5.44 ± 0.05 ^aD^	5.60 ± 0.02 ^aCD^	5.71 ± 0.08 ^bcC^	6.10 ± 0.11 ^abB^	6.11 ± 0.15 ^bcdB^	6.38 ± 0.03 ^dA^
A2.5	5.44 ± 0.05 ^aC^	5.58 ± 0.01 ^aBC^	5.61 ± 0.09 ^bcBC^	5.89 ± 0.10 ^bcB^	6.43 ± 0.29 ^abA^	6.60 ± 0.08 ^bcA^
G2.5	5.44 ± 0.05 ^aD^	5.55 ± 0.01 ^abcCD^	5.76 ± 0.02 ^abcC^	6.22 ± 0.11 ^aB^	6.35 ± 0.21 ^abB^	6.75 ± 0.05 ^bA^
A5	5.44 ± 0.05 ^aD^	5.57 ± 0.01 ^abD^	5.97 ± 0.16 ^aC^	5.97 ± 0.04 ^abcC^	6.30 ± 0.07 ^abB^	6.62 ± 0.05 ^bcA^
G5	5.44 ± 0.05 ^aE^	5.51 ± 0.03 ^cE^	5.76 ± 0.05 ^abcD^	6.13 ± 0.10 ^abC^	6.59 ± 0.11 ^aB^	7.00 ± 0.07 ^aA^
C−	5.44 ± 0.05 ^aD^	5.57 ± 0.02 ^abCD^	5.84 ± 0.11 ^abC^	6.22 ± 0.15 ^aB^	6.26 ± 0.16 ^abcAB^	6.54 ± 0.05 ^cdA^
C+	5.50 ± 0.03 ^aC^	5.57 ± 0.02 ^aC^	5.72 ± 0.01 ^bcB^	5.72 ± 0.03 ^cB^	5.79 ± 0.04 ^dB^	6.03 ± 0.07 ^eA^

* (C−) negative control, patty without additives; (C+) positive control, patty with commercial preparation; (A5) patty covered in 5% extract-based alginate film; (A2.5) patty covered in 2.5% extract-based alginate film; (WA) patty covered in water-based alginate film; (G5). ^A–E^ Mean values with different superscripts within the same treatment but at different times are statistically different. ^a–e^ Mean values with different superscripts on the same day but from different treatments are statistically different (*p* < 0.05).

**Table 4:** Variations in fatty acid percentages in patties with eight different treatments.

Fatty Acid	Day 0	Day 7							
(%)	C−	C−	WA	WG	A2.5	G2.5	A5	G5	C+
C12:0	0.07 ± 0.00 ^c^	0.07 ± 0.00 ^c^	0.15 ± 0.01 ^a^	0.09 ± 0.09 ^b^	0.07 ± 0.00 ^c^	0.06 ± 0.00 ^d^	0.07 ± 0.06 ^c^	0.07 ± 0.05 ^c^	0.07 ± 0.00 ^c^
C14:0	3.11 ± 0.01 ^f^	3.93 ± 0.05 ^b^	3.08 ± 0.00 ^g^	1.53 ± 0.04 ^h^	3.11 ± 0.02 ^f^	4.37 ± 0.00 ^a^	3.37 ± 0.01 ^d^	3.46 ± 0.00 ^c^	3.26 ± 0.05 ^e^
C15:0	0.49 ± 0.01 ^d^	0.50 ± 0.01 ^c^	0.47 ± 0.01 ^f^	0.48 ± 0.00 ^e^	0.47 ± 0.00 ^f^	0.66 ± 0.00 ^a^	0.51 ± 0.14 ^b^	0.50 ± 0.00 ^c^	0.50 ± 0.05 ^c^
C16:0	30.06 ± 0.01 ^f^	30.54 ± 0.06 ^b^	28.93 ± 0.00 ^h^	30.21 ± 0.02 ^d^	29.20 ± 0.02 ^g^	28.71 ± 0.01 ^i^	30.75 ± 0.02 ^a^	30.23 ± 0.00 ^c^	30.13 ± 0.06 ^e^
C17:0	1.37 ± 0.59 ^a^	1.30 ± 0.02 ^d^	1.36 ± 0.00 ^b^	1.30 ± 0.14 ^d^	1.31 ± 0.00 ^c^	1.30 ± 0.28 ^d^	1.30 ± 0.06 ^d^	1.26 ± 0.22 ^e^	1.31 ± 0.68 ^c^
C18:0	14.10 ± 0.02 ^a^	13.74 ± 0.18 ^c^	13.88 ± 0.01 ^b^	13.18 ± 0.16 ^g^	12.99 ± 0.04 ^i^	13.06 ± 0.00 ^h^	13.22 ± 0.00 ^f^	13.69 ± 0.22 ^d^	13.61 ± 0.18 ^e^
C20:0	0.08 ± 0.00 ^f^	0.09 ± 0.00 ^e^	0.23 ± 0.01 ^a^	0.18 ± 0.00 ^b^	0.12 ± 0.00 ^c^	0.07 ± 0.00 ^g^	0.10 ± 0.00 ^d^	0.07 ± 0.00 ^g^	0.06 ± 0.00 ^h^
C21:0	0.17 ± 0.00 ^c^	0.15 ± 0.00 ^e^	0.14 ± 0.00 ^f^	0.15 ± 0.00 ^e^	0.07 ± 0.02 ^g^	0.22 ± 0.00 ^a^	0.16 ± 0.00 ^d^	0.14 ± 0.01 ^f^	0.18 ± 0.00 ^b^
C22:0	0.11 ± 0.01 ^a^	0.04 ± 0.00 ^e^	ND	ND	ND	0.09 ± 0.07 ^b^	0.08 ± 0.00 ^c^	0.07 ± 0.00 ^f^	0.08 ± 0.00 ^c^
C14:1	0.79 ± 0.00 ^g^	0.87 ± 0.01 ^c^	0.75 ± 0.00 ^i^	0.84 ± 0.00 ^e^	0.81 ± 0.01 ^f^	0.76 ± 0.00 ^h^	0.92 ± 0.00 ^a^	0.89 ± 0.00 ^b^	0.85 ± 0.01 ^d^
C15:1	0.09 ± 0.03 ^b^	0.09 ± 0.00 ^b^	0.08 ± 0.01 ^c^	0.07 ± 0.00 ^d^	0.79 ± 0.07 ^a^	0.04 ± 0.00 ^e^	0.09 ± 0.02 ^b^	0.09 ± 0.00 ^b^	0.01 ± 0.07 ^f^
C16:1	3.39 ± 0.03 ^g^	3.81 ± 0.08 ^b^	3.24 ± 0.01 ^i^	3.75 ± 0.02 ^c^	3.51 ± 0.06 ^f^	3.26 ± 0.03 ^h^	3.82 ± 0.04 ^a^	3.54 ± 0.05 ^e^	3.60 ± 0.08 ^d^
C17:1	1.01 ± 0.03 ^a^	0.97 ± 0.08 ^d^	1.00 ± 0.01 ^b^	1.01 ± 0.06 ^a^	1.00 ± 0.06 ^b^	0.99 ± 0.04 ^c^	0.70 ± 0.05 ^f^	0.90 ± 0.00 ^e^	1.00 ± 0.08 ^b^
C18:1 trans	ND	0.25 ± 0.03 ^f^	3.12 ± 0.01 ^c^	3.06 ± 0.03 ^d^	3.41 ± 0.03 ^b^	1.98 ± 0.03 ^e^	0.15 ± 0.03 ^g^	3.91 ± 0.03 ^a^	ND
C18:1 cis	42.53 ± 1.29 ^b^	42.03 ± 2.50 ^e^	41.36 ± 0.01 ^g^	42.05 ± 2.50 ^d^	41.89 ± 0.62 ^f^	41.20 ± 0.83 ^h^	42.35 ± 0.19 ^c^	38.94 ± 0.52 ^i^	42.94 ± 2.50 ^a^
C20:1	0.07 ± 0.01 ^b^	0.06 ± 0.08 ^c^	ND	ND	ND	0.07 ± 0.02 ^b^	0.08 ± 0.00 ^a^	0.07 ± 0.00 ^b^	0.07 ± 0.08 ^b^
C20:3	ND	0.05 ± 0.08 ^d^	0.12 ± 0.01 ^b^	0.12 ± 0.07 ^b^	0.10 ± 0.06 ^c^	ND	0.13 ± 0.00 ^a^	0.03 ± 0.00 ^e^	ND
C18:2 cis	2.03 ± 0.71 ^b^	1.63 ± 0.01 ^e^	1.60 ± 0.01 ^f^	1.65 ± 0.07 ^d^	1.65 ± 0.06 ^d^	2.33 ± 0.00 ^a^	1.72 ± 0.01 ^c^	1.59 ± 0.33 ^g^	1.72 ± 0.88 ^c^
C18:2 trans	0.09 ± 0.02 ^c^	0.07 ± 0.08 ^d^	0.05 ± 0.01 ^f^	0.06 ± 0.06 ^e^	0.06 ± 0.03 ^e^	0.14 ± 0.00 ^a^	0.09 ± 0.01 ^c^	0.10 ± 0.00 ^b^	0.10 ± 0.00 ^b^
C18:3 ga	0.08 ± 0.02 ^c^	0.06 ± 0.08 ^d^	0.23 ± 0.01 ^a^	ND	0.04 ± 0.00 ^f^	0.10 ± 0.01 ^b^	0.10 ± 0.01 ^b^	0.05 ± 0.00 ^e^	0.04 ± 0.00 ^f^
C18:3 alph	0.24 ± 0.02 ^c^	0.24 ± 0.08 ^c^	0.22 ± 0.01 ^e^	0.23 ± 0.00 ^d^	ND	0.32 ± 0.01 ^a^	0.22 ± 0.01 ^e^	0.23 ± 0.02 ^d^	0.25 ± 0.00 ^b^
C20:2 n-6	ND	ND	ND	ND	ND	ND	0.01 ± 0.00 ^a^	0.01 ± 0.00 ^a^	ND
C20:5 n-3(EPA)	0.11 ± 0.01 ^b^	0.04 ± 0.08 ^g^	0.18 ± 0.01 ^a^	ND	0.06 ± 0.00 ^e^	0.08 ± 0.41 ^d^	0.10 ± 0.01 ^c^	0.05 ± 0.00 ^f^	0.06 ± 0.00 ^e^
SFA	49.57 ^b^	50.36 ^a^	48.39 ^f^	47.13 ^h^	47.35 ^g^	48.54 ^e^	49.56 ^b^	49.51 ^c^	49.22 ^d^
MUFA	47.90 ^g^	48.13 ^f^	49.68 ^b^	50.91 ^a^	50.81 ^a^	48.50 ^cd^	48.25 ^ef^	48.38 ^de^	48.58 ^c^
PUFA	2.53 ^b^	2.04 ^g^	2.28 ^c^	1.95 ^h^	1.77 ^i^	2.95 ^a^	2.18 ^e^	2.10 ^f^	2.19 ^d^
MUFA + PUFA/SFA	1.01	1.00	1.07	1.12	1.11	1.06	1.02	1.02	1.03

(C−) negative control, patty without additives; (CP) positive control, patty with commercial preparation; (A5) patty covered in 5% extract-based alginate film; (A25) patty covered in 2.5% extract-based alginate film; (WA) patty covered in water-based alginate film; (G5) patty covered in 5% extract-based gelatin film; (G25) patty covered in 2.5% extract-based gelatin film; (WG) patty covered in water-based gelatin film. Acronyms: SFA—saturated fatty acids; MUFA—monounsaturated fatty acids; PUFA—polyunsaturated fatty acids; C12:0 (lauric acid), C14:0 (myristic acid), C15:0 (pentadecanoic acid), C16:0 (palmitic acid), C17:0 (heptadecanoic acid), C18:0 (stearic acid), C20:0 (arachidic acid), C21:0 (heneicosanoic acid), C22:0 (behenic acid), C14:1 (myristoleic acid), C15:1 (pentadecenoic acid), C16:1 (palmitoleic acid), C17:1 (heptadecenoic acid), C18:1 trans (elaidic acid, trans isomer of oleic), C18:1 cis (oleic acid), C20:1 (eicosenoic acid), C20:3 (eicosatrienoic acid), C18:2 cis (linoleic acid), C18:2 trans (linolelaidic acid), C18:3 ga (γ-linolenic acid, omega-6), C18:3 alph (α-linolenic acid, omega-3), C20:2 n-6 (eicosadienoic acid) and C20:5 n-3 (EPA, eicosapentaenoic acid). ^a–i^ Mean values with different superscripts in a row indicate a significant (*p* < 0.05) difference between the different treatments at the same fatty acid level. The experimental data were expressed as mean ± standard error (SE).

## Data Availability

The original contributions presented in the study are included in the article, further inquiries can be directed to the corresponding authors.
